# Crystal structure of *N*-(4-chloro­phen­yl)benzo­thio­amide

**DOI:** 10.1107/S2056989015008075

**Published:** 2015-04-30

**Authors:** Ganlin Zhao

**Affiliations:** a Changsha Environmental Protection College, Changsha 410004, People’s Republic of China

**Keywords:** crystal structure, benzo­thio­amide, N—H⋯S hydrogen bonding.

## Abstract

The title compound, C_13_H_10_ClNS, exhibits a *trans* conformation with regard to the axis of the C—N bond. The benzene and phenyl rings are inclined to one another by 85.06 (8)°. In the crystal, mol­ecules are linked by N—H⋯S=C hydrogen bonds, forming chains along [001].

## Related literature   

For hydrogen bonding of amides, see: Taylor *et al.* (1984 ▸); Leiserowitz & Schmidt (1969 ▸). For the preparation and for the use of thio­amides as inter­mediates in chemical transformations, see: Li *et al.* (2012 ▸, 2015 ▸). For related structures, see: Omondi *et al.* (2012 ▸); Nagasawa *et al.* (2014 ▸).
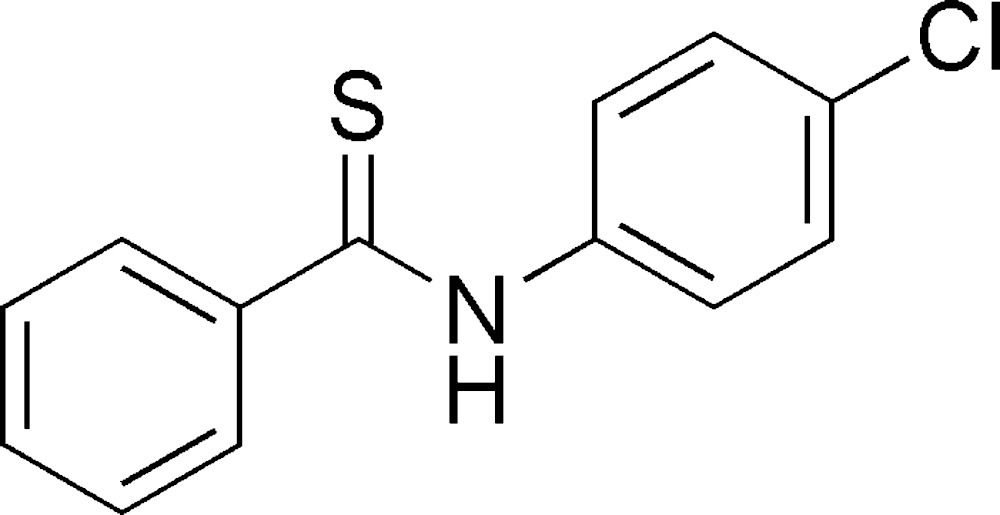


## Experimental   

### Crystal data  

C_13_H_10_ClNS
*M* *_r_* = 247.73
Monoclinic, 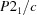

*a* = 11.943 (2) Å
*b* = 12.689 (3) Å
*c* = 7.9764 (16) Å
β = 109.30 (3)°
*V* = 1140.9 (4) Å^3^
*Z* = 4
Mo *K*α radiation
μ = 0.49 mm^−1^
*T* = 113 K
0.22 × 0.20 × 0.12 mm

### Data collection  

Rigaku Saturn CCD area-detector diffractometer
Absorption correction: multi-scan (*CrystalClear*; Rigaku/MSC, 2005 ▸) *T*_min_ = 0.901, *T*_max_ = 0.944
7517 measured reflections
2010 independent reflections
1701 reflections with *I* > 2σ(*I*)
*R*_int_ = 0.030

### Refinement  

*R*[*F* ^2^ > 2σ(*F* ^2^)] = 0.028
*wR*(*F* ^2^) = 0.077
*S* = 1.05
2010 reflections
150 parameters
1 restraint
H atoms treated by a mixture of independent and constrained refinement
Δρ_max_ = 0.22 e Å^−3^
Δρ_min_ = −0.18 e Å^−3^

### 

Data collection: *CrystalClear* (Rigaku/MSC, 2005 ▸); cell refinement: *CrystalClear*; data reduction: *CrystalClear*; program(s) used to solve structure: *SHELXS97* (Sheldrick, 2008 ▸); program(s) used to refine structure: *SHELXL97* (Sheldrick, 2008 ▸); molecular graphics: *SHELXTL* (Sheldrick, 2008 ▸); software used to prepare material for publication: *SHELXL97*.

## Supplementary Material

Crystal structure: contains datablock(s) I. DOI: 10.1107/S2056989015008075/su5123sup1.cif

Structure factors: contains datablock(s) I. DOI: 10.1107/S2056989015008075/su5123Isup2.hkl

Supporting information file. DOI: 10.1107/S2056989015008075/su5123Isup3.cml

**Fig. 1.**
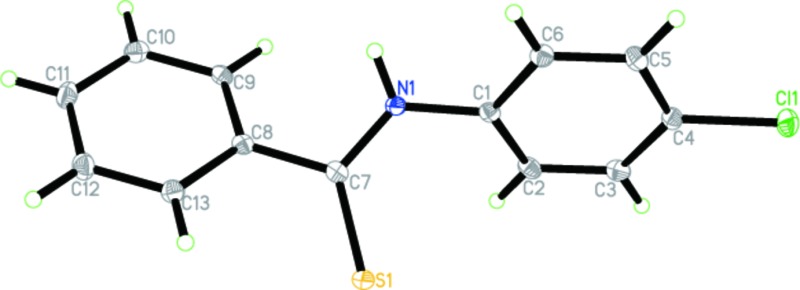
The molecular structure of the title compound, with atom labelling. Displacement ellipsoids are drawn at the 30% probability level.

**Fig. 2.**
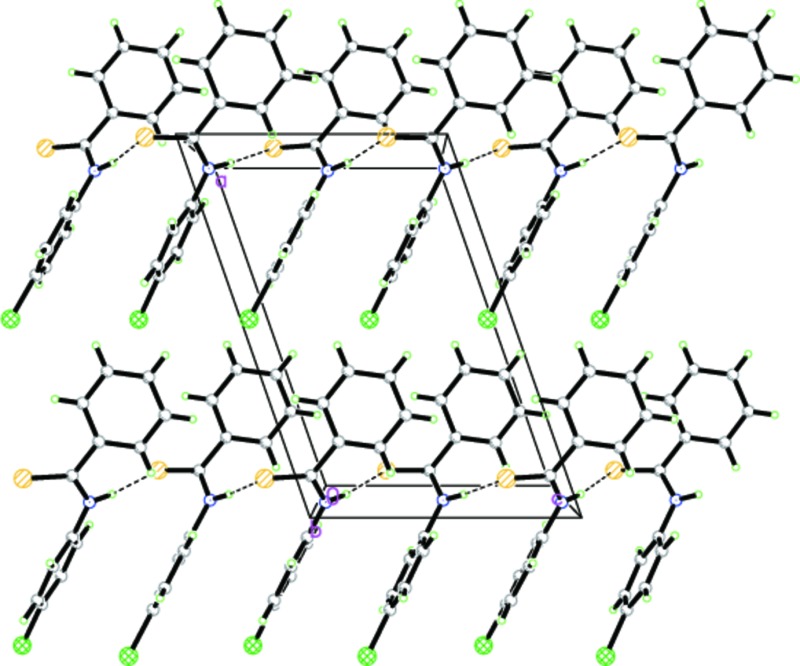
A view along the b axis of the crystal packing of the title compound. The N—H···S hydrogen bonds are shown as dashed lines (see Table 1 for details).

. DOI: 10.1107/S2056989015008075/su5123fig1.tif
The mol­ecular structure of the title compound, with atom labelling. Displacement ellipsoids are drawn at the 30% probability level.

b . DOI: 10.1107/S2056989015008075/su5123fig2.tif
A view along the *b* axis of the crystal packing of the title compound. The N—H⋯S hydrogen bonds are shown as dashed lines (see Table 1 for details).

CCDC reference: 1061304

Additional supporting information:  crystallographic information; 3D view; checkCIF report

## Figures and Tables

**Table 1:** Hydrogen-bond geometry (, )

*D*H*A*	*D*H	H*A*	*D* *A*	*D*H*A*
N1H1S1^i^	0.89(1)	2.49(1)	3.346(15)	163(1)

Symmetry code: (i) 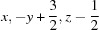.

## supplementary crystallographic information

### S1. Comment

Thioamides are extensively used as extractant for heavy metals in environmental chemistry, as intermediate for important chemical transformations (Li *et al.*, 2015), and also used to replace the amide bonds as isosteres. It's well known that amide units can be connected by a N—H···O=C hydrogen bonds (Taylor *et al.*, 1984; Leiserowitz & Schmidt, 1969), and also the structures of some thioamides have been documented (Omondi *et al.*, 2012; Nagasawa *et al.*, 2014). Herein, we report the crystal structure of the title compound (I).

Figure 1 has shows the molecular structure of the title compound, whose thioamide unit adopts a *trans* conformation around the central C-N bond. The C=S double bond is deviated from its connected phenyl ring [torsion angles: S1/C7/C8/C9 -145.34 (13)°,S1/C7/C8/C13 33.62 (19)°]. The benzene and phenyl rings are inclined to one another by 85.06 (8) °.

In the crystal, molecules are linked *via* N—H···S═C hydrogen bonds, forming chains along the *c* axis direction (Table 1 and Fig. 2).

### S2. Experimental

The title compound was prepared from the Beckmann rearrangement from its corresponding ketoximes following a published procedure (Li *et al.*, 2012). It was isolated by flash chromatography and yellow block-like crystal of the title compound were obtained *via* natural evaporation from the diluent.

### S3. Refinement

The thioamide N—H atom was located in a difference Fourier map and freely refined. The C-bound H atoms were included in calculated positions and refined as riding atoms: C—H = 0.93 Å with *U*_iso_(H) = 1.2 *U*_eq_(C).

### Crystal data

**Table:**

C_13_H_10_ClNS	*F*(000) = 512
*M**_r_* = 247.73	*D*_x_ = 1.442 Mg m^−^^3^
Monoclinic, *P*2_1_/*c*	Mo *K*α radiation, λ = 0.71073 Å
Hall symbol: -P 2ybc	Cell parameters from 3499 reflections
*a* = 11.943 (2) Å	θ = 1.8–27.9°
*b* = 12.689 (3) Å	µ = 0.49 mm^−^^1^
*c* = 7.9764 (16) Å	*T* = 113 K
β = 109.30 (3)°	Block, yellow
*V* = 1140.9 (4) Å^3^	0.22 × 0.20 × 0.12 mm
*Z* = 4	

### Data collection

**Table:**

Rigaku Saturn CCD area-detector diffractometer	2010 independent reflections
Radiation source: rotating anode	1701 reflections with *I* > 2σ(*I*)
Confocal monochromator	*R*_int_ = 0.030
Detector resolution: 7.31 pixels mm^-1^	θ_max_ = 25.0°, θ_min_ = 2.4°
ω and φ scans	*h* = −11→14
Absorption correction: multi-scan (*CrystalClear*; Rigaku/MSC, 2005)	*k* = −15→15
*T*_min_ = 0.901, *T*_max_ = 0.944	*l* = −9→9
7517 measured reflections	

### Refinement

**Table:**

Refinement on *F*^2^	Secondary atom site location: difference Fourier map
Least-squares matrix: full	Hydrogen site location: inferred from neighbouring sites
*R*[*F*^2^ > 2σ(*F*^2^)] = 0.028	H atoms treated by a mixture of independent and constrained refinement
*wR*(*F*^2^) = 0.077	*w* = 1/[σ^2^(*F*_o_^2^) + (0.0477*P*)^2^ + 0.1743*P*] where *P* = (*F*_o_^2^ + 2*F*_c_^2^)/3
*S* = 1.05	(Δ/σ)_max_ = 0.002
2010 reflections	Δρ_max_ = 0.22 e Å^−^^3^
150 parameters	Δρ_min_ = −0.18 e Å^−^^3^
1 restraint	Extinction correction: *SHELXL97* (Sheldrick, 2008), Fc^*^=kFc[1+0.001xFc^2^λ^3^/sin(2θ)]^-1/4^
Primary atom site location: structure-invariant direct methods	Extinction coefficient: 0.050 (4)

### Special details

**Table:**

Geometry. All e.s.d.'s (except the e.s.d. in the dihedral angle between two l.s. planes) are estimated using the full covariance matrix. The cell e.s.d.'s are taken into account individually in the estimation of e.s.d.'s in distances, angles and torsion angles; correlations between e.s.d.'s in cell parameters are only used when they are defined by crystal symmetry. An approximate (isotropic) treatment of cell e.s.d.'s is used for estimating e.s.d.'s involving l.s. planes.
Refinement. Refinement of *F*^2^ against ALL reflections. The weighted *R*-factor *wR* and goodness of fit *S* are based on *F*^2^, conventional *R*-factors *R* are based on *F*, with *F* set to zero for negative *F*^2^. The threshold expression of *F*^2^ > σ(*F*^2^) is used only for calculating *R*-factors(gt) *etc*. and is not relevant to the choice of reflections for refinement. *R*-factors based on *F*^2^ are statistically about twice as large as those based on *F*, and *R*- factors based on ALL data will be even larger.

### Fractional atomic coordinates and isotropic or equivalent isotropic displacement parameters (Å^2^)

**Table:**

	*x*	*y*	*z*	*U*_iso_*/*U*_eq_	
S1	0.94504 (4)	0.58973 (3)	1.20150 (5)	0.01919 (16)	
Cl1	1.47747 (4)	0.84240 (4)	1.54329 (6)	0.02760 (17)	
N1	1.02619 (11)	0.71403 (10)	0.99722 (17)	0.0160 (3)	
C1	1.13474 (13)	0.74209 (12)	1.1311 (2)	0.0159 (3)	
C2	1.21244 (14)	0.66609 (13)	1.2321 (2)	0.0201 (4)	
H2	1.1936	0.5949	1.2147	0.024*	
C3	1.31777 (14)	0.69722 (13)	1.3583 (2)	0.0218 (4)	
H3	1.3698	0.6470	1.4264	0.026*	
C4	1.34551 (14)	0.80302 (13)	1.3828 (2)	0.0186 (4)	
C5	1.26984 (15)	0.87901 (13)	1.2816 (2)	0.0219 (4)	
H5	1.2895	0.9501	1.2982	0.026*	
C6	1.16448 (15)	0.84782 (13)	1.1550 (2)	0.0194 (4)	
H6	1.1134	0.8982	1.0857	0.023*	
C7	0.94159 (14)	0.64895 (12)	1.0128 (2)	0.0159 (4)	
C8	0.83987 (14)	0.63239 (12)	0.8467 (2)	0.0161 (4)	
C9	0.85648 (15)	0.62942 (12)	0.6821 (2)	0.0182 (4)	
H9	0.9320	0.6389	0.6751	0.022*	
C10	0.76076 (16)	0.61237 (13)	0.5280 (2)	0.0226 (4)	
H10	0.7725	0.6106	0.4184	0.027*	
C11	0.64826 (16)	0.59810 (13)	0.5376 (2)	0.0249 (4)	
H11	0.5842	0.5868	0.4347	0.030*	
C12	0.63130 (15)	0.60079 (13)	0.7015 (2)	0.0251 (4)	
H12	0.5557	0.5910	0.7082	0.030*	
C13	0.72593 (14)	0.61784 (12)	0.8545 (2)	0.0201 (4)	
H13	0.7137	0.6197	0.9637	0.024*	
H1	1.0058 (16)	0.7552 (13)	0.9013 (17)	0.030 (5)*	

### Atomic displacement parameters (Å^2^)

**Table:**

	*U*^11^	*U*^22^	*U*^33^	*U*^12^	*U*^13^	*U*^23^
S1	0.0205 (2)	0.0192 (2)	0.0179 (3)	−0.00251 (17)	0.00650 (18)	0.00248 (16)
Cl1	0.0174 (2)	0.0353 (3)	0.0268 (3)	−0.00442 (18)	0.00281 (18)	−0.00048 (18)
N1	0.0177 (7)	0.0151 (7)	0.0155 (7)	−0.0010 (6)	0.0057 (6)	0.0010 (6)
C1	0.0158 (8)	0.0181 (8)	0.0164 (8)	−0.0003 (7)	0.0090 (7)	−0.0010 (6)
C2	0.0187 (9)	0.0154 (8)	0.0280 (9)	0.0004 (7)	0.0102 (7)	0.0010 (7)
C3	0.0170 (8)	0.0215 (9)	0.0279 (10)	0.0042 (7)	0.0089 (7)	0.0066 (7)
C4	0.0138 (8)	0.0245 (9)	0.0186 (8)	−0.0014 (7)	0.0070 (7)	−0.0008 (7)
C5	0.0231 (9)	0.0177 (8)	0.0249 (9)	−0.0026 (7)	0.0081 (8)	−0.0024 (7)
C6	0.0192 (9)	0.0175 (8)	0.0211 (9)	0.0029 (7)	0.0063 (7)	0.0016 (6)
C7	0.0173 (8)	0.0118 (7)	0.0210 (9)	0.0028 (6)	0.0096 (7)	−0.0021 (6)
C8	0.0186 (8)	0.0098 (7)	0.0201 (8)	0.0007 (6)	0.0065 (7)	−0.0005 (6)
C9	0.0202 (9)	0.0133 (8)	0.0223 (9)	−0.0018 (7)	0.0085 (7)	0.0000 (7)
C10	0.0300 (10)	0.0194 (8)	0.0186 (9)	−0.0017 (8)	0.0081 (8)	−0.0018 (7)
C11	0.0234 (9)	0.0257 (9)	0.0202 (9)	−0.0010 (8)	0.0001 (7)	−0.0017 (7)
C12	0.0171 (9)	0.0280 (9)	0.0285 (10)	0.0010 (8)	0.0052 (7)	0.0009 (8)
C13	0.0192 (9)	0.0210 (9)	0.0212 (9)	0.0019 (7)	0.0080 (7)	0.0011 (7)

### Geometric parameters (Å, º)

**Table:**

S1—C7	1.6705 (16)	C6—H6	0.9300
Cl1—C4	1.7430 (17)	C7—C8	1.487 (2)
N1—C7	1.342 (2)	C8—C9	1.391 (2)
N1—C1	1.426 (2)	C8—C13	1.395 (2)
N1—H1	0.891 (9)	C9—C10	1.392 (2)
C1—C6	1.385 (2)	C9—H9	0.9300
C1—C2	1.395 (2)	C10—C11	1.383 (2)
C2—C3	1.384 (2)	C10—H10	0.9300
C2—H2	0.9300	C11—C12	1.388 (3)
C3—C4	1.381 (2)	C11—H11	0.9300
C3—H3	0.9300	C12—C13	1.379 (2)
C4—C5	1.384 (2)	C12—H12	0.9300
C5—C6	1.385 (2)	C13—H13	0.9300
C5—H5	0.9300		
			
C7—N1—C1	127.60 (13)	N1—C7—C8	115.00 (13)
C7—N1—H1	116.1 (12)	N1—C7—S1	124.40 (13)
C1—N1—H1	114.8 (12)	C8—C7—S1	120.59 (12)
C6—C1—C2	119.92 (15)	C9—C8—C13	118.95 (15)
C6—C1—N1	118.25 (14)	C9—C8—C7	121.01 (15)
C2—C1—N1	121.77 (14)	C13—C8—C7	120.03 (15)
C3—C2—C1	119.60 (15)	C8—C9—C10	120.42 (16)
C3—C2—H2	120.2	C8—C9—H9	119.8
C1—C2—H2	120.2	C10—C9—H9	119.8
C4—C3—C2	119.90 (15)	C11—C10—C9	120.03 (16)
C4—C3—H3	120.0	C11—C10—H10	120.0
C2—C3—H3	120.0	C9—C10—H10	120.0
C3—C4—C5	120.96 (15)	C10—C11—C12	119.76 (16)
C3—C4—Cl1	119.94 (13)	C10—C11—H11	120.1
C5—C4—Cl1	119.09 (13)	C12—C11—H11	120.1
C4—C5—C6	119.13 (15)	C13—C12—C11	120.34 (16)
C4—C5—H5	120.4	C13—C12—H12	119.8
C6—C5—H5	120.4	C11—C12—H12	119.8
C1—C6—C5	120.46 (15)	C12—C13—C8	120.50 (16)
C1—C6—H6	119.8	C12—C13—H13	119.7
C5—C6—H6	119.8	C8—C13—H13	119.7
			
C7—N1—C1—C6	−131.37 (16)	C1—N1—C7—S1	2.6 (2)
C7—N1—C1—C2	51.1 (2)	N1—C7—C8—C9	35.2 (2)
C6—C1—C2—C3	1.3 (2)	S1—C7—C8—C9	−145.34 (13)
N1—C1—C2—C3	178.80 (14)	N1—C7—C8—C13	−145.86 (15)
C1—C2—C3—C4	−0.3 (2)	S1—C7—C8—C13	33.62 (19)
C2—C3—C4—C5	−0.7 (2)	C13—C8—C9—C10	0.1 (2)
C2—C3—C4—Cl1	179.35 (12)	C7—C8—C9—C10	179.08 (14)
C3—C4—C5—C6	0.5 (2)	C8—C9—C10—C11	−0.1 (2)
Cl1—C4—C5—C6	−179.46 (12)	C9—C10—C11—C12	0.0 (2)
C2—C1—C6—C5	−1.5 (2)	C10—C11—C12—C13	0.2 (3)
N1—C1—C6—C5	−179.01 (14)	C11—C12—C13—C8	−0.1 (2)
C4—C5—C6—C1	0.5 (2)	C9—C8—C13—C12	0.0 (2)
C1—N1—C7—C8	−177.96 (13)	C7—C8—C13—C12	−178.98 (14)

### Hydrogen-bond geometry (Å, º)

**Table:**

*D*—H···*A*	*D*—H	H···*A*	*D*···*A*	*D*—H···*A*
N1—H1···S1^i^	0.89 (1)	2.49 (1)	3.346 (15)	163 (1)

Symmetry code: (i) *x*, −*y*+3/2, *z*−1/2.
