# Supplementary figures and images for: Crystal structure of N-(4-chloro­phen­yl)benzo­thio­amide
Source: Acta Crystallogr E Crystallogr Commun. 2015 Apr 30;71(Pt 5):o353. doi: 10.1107/S2056989015008075 (PMC4420071; doi:10.1107/S2056989015008075)

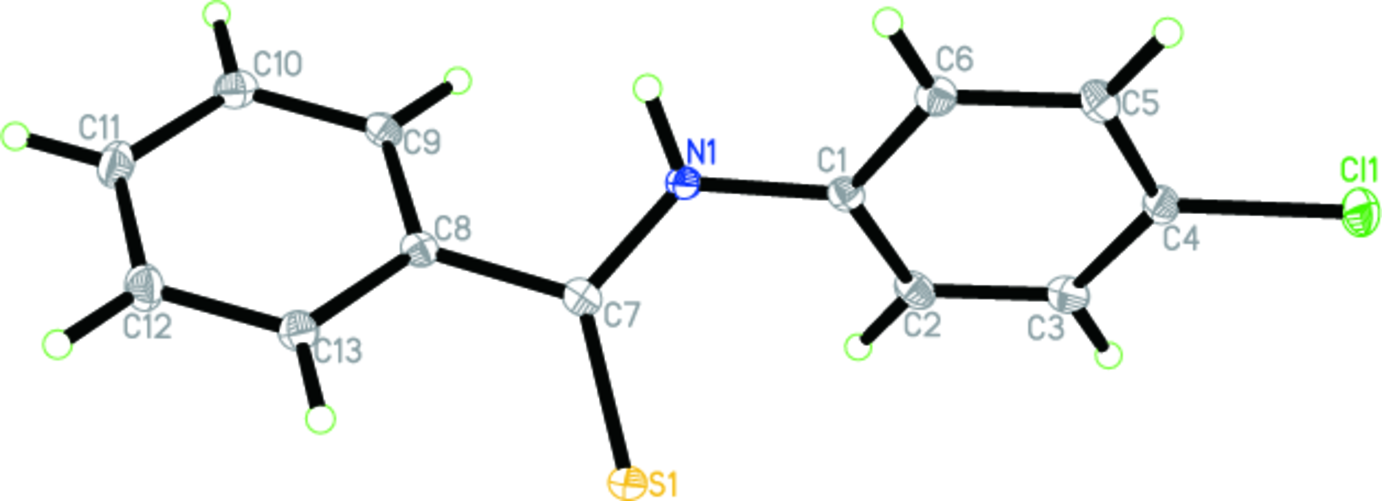

Supplement: Supplementary file 4 [file e-71-0o353-fig1.tif]

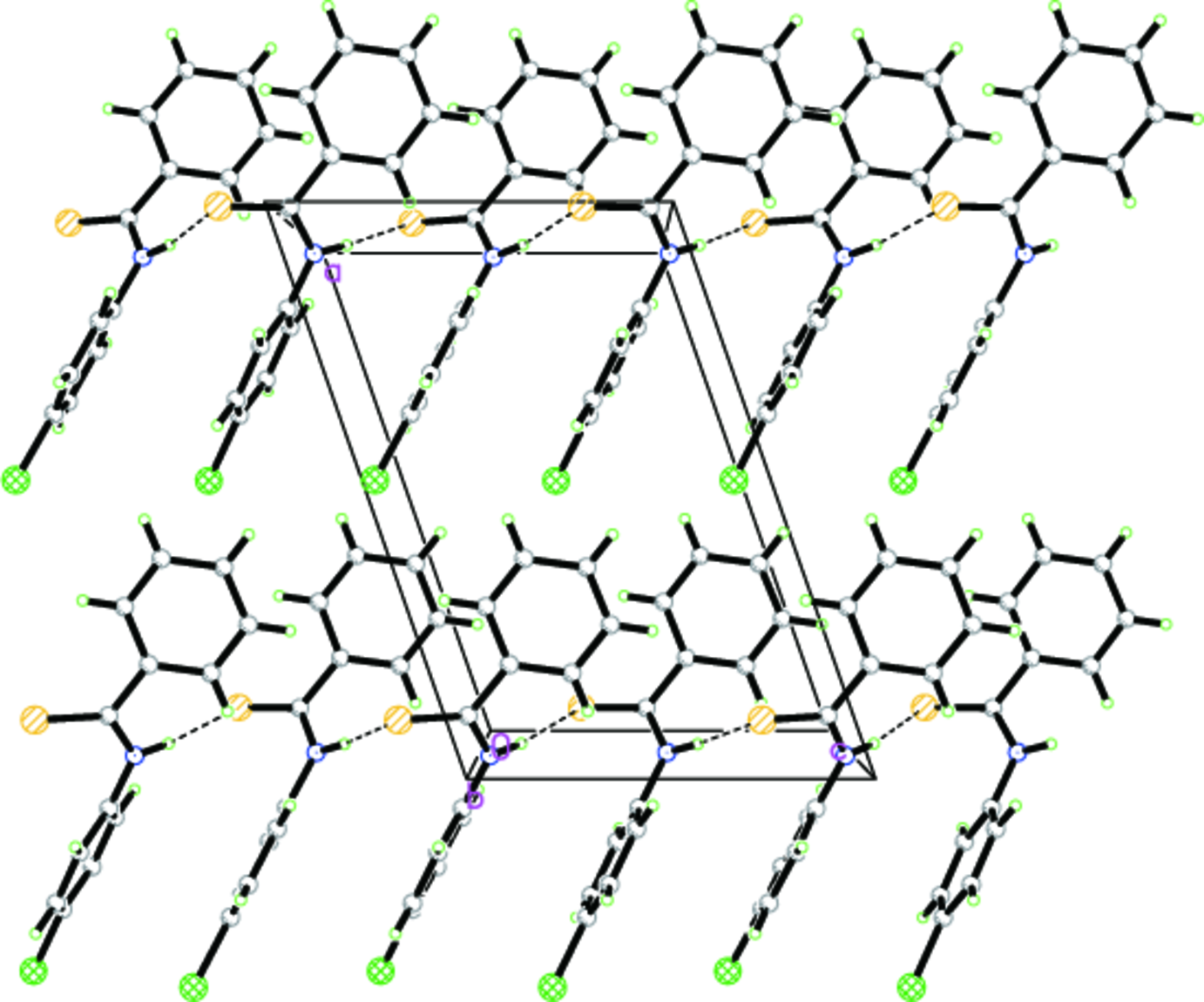

Supplement: Supplementary file 5 [file e-71-0o353-fig2.tif]
